# First insight into the faecal microbiota of the high Arctic muskoxen (*Ovibos moschatus*)

**DOI:** 10.1099/mgen.0.000066

**Published:** 2016-07-29

**Authors:** Alejandro Salgado-Flores, Mathias Bockwoldt, Live H. Hagen, Phillip B. Pope, Monica A. Sundset

**Affiliations:** ^1^​ University of Tromsø, Tromso 9019, Norway; ^2^​ Department of Arctic and Marine Biology, UiT – The Arctic University of Norway, Tromsø, Norway; ^3^​ Department of Chemistry, Biotechnology and Food Science, Norwegian University of Life Sciences, Ås, Norway

**Keywords:** ruminant faeces, 16S rRNA, *Archaea*, *Bacteria*, methanogens, pyrosequencing

## Abstract

The faecal microbiota of muskoxen (*n*=3) pasturing on Ryøya (69° 33′ N 18° 43′ E), Norway, in late September was characterized using high-throughput sequencing of partial 16S rRNA gene regions. A total of 16 209 high-quality sequence reads from bacterial domains and 19 462 from archaea were generated. Preliminary taxonomic classifications of 806 bacterial operational taxonomic units (OTUs) resulted in 53.7–59.3 % of the total sequences being without designations beyond the family level. *Firmicutes* (70.7–81.1 % of the total sequences) and *Bacteroidetes* (16.8–25.3 %) constituted the two major bacterial phyla, with uncharacterized members within the family *Ruminococcaceae* (28.9–40.9 %) as the major phylotype. Multiple-library comparisons between muskoxen and other ruminants indicated a higher similarity for muskoxen faeces and reindeer caecum (*P*>0.05) and some samples from cattle faeces. The archaeal sequences clustered into 37 OTUs, with dominating phylotypes affiliated to the methane-producing genus *Methanobrevibacter* (80–92 % of the total sequences). UniFrac analysis demonstrated heterogeneity between muskoxen archaeal libraries and those from reindeer and roe deer (*P*=1.0e-02, Bonferroni corrected), but not with foregut fermenters. The high proportion of cellulose-degrading *Ruminococcus*-affiliated bacteria agrees with the ingestion of a highly fibrous diet. Further experiments are required to elucidate the role played by these novel bacteria in the digestion of this fibrous Artic diet eaten by muskoxen.

## Data Summary

1. 16S rRNA bacterial and archaeal sequences used in the study have been deposited in the Sequence Read Archive (Bioproject: SRP049372) (url - http://www.ncbi.nlm.nih.gov/sra/?term=SRP049372)

## Impact Statement

This study gives a first glimpse into the faecal bacterial and archaeal microbiota harboured in faeces from muskoxen, giving a broad view of this highly complex microbial environment. Only one previous study has been conducted on the gut microbiome of muskoxen, describing their rumen eukaryotes. The results presented here are consequently an interesting complement to gain further insight into the microbial diversity housed in the gastrointestinal tract of muskoxen. The relatively high percentage of novel bacterial and archaeal phylotypes in the current study points to a need for new experiments to identify this putatively novel microbiota and their related functional gene contents. Taken together, the results presented here not only help increase knowledge on the microbial diversity occurring with highly fibrous diets but also on the microbiological aspects related to methanogenesis in Arctic ruminants.

## Introduction

Muskoxen (*Ovibos moschatus*) are one of few large, terrestrial mammals adapted to a high Arctic environment. It was once holarctically distributed, but became extinct over most of its Eurasian range at the beginning of the Holocene, and is currently found as relict natural populations mainly in Greenland, northern Canada and Alaska (Campos *et al.*, 2010). In their natural habitat, muskoxen are typical grass and roughage eaters, feeding on grass heath communities or willows found on exposed ridges, slopes or plains. Their anatomy consists of a large rumen–reticulum (containing 34–78 % of the total alimentary tract contents), a large omasum and a relatively small caecal–colon complex, all contributing to extremely long retention times (Thing *et al.*, 1987; Staaland & Thing, 1991; Staaland *et al.*, 1997). For most of the year the animals graze on forages high in lignocellulose, whereas high-quality forages are only available during the short Arctic summer (Thing *et al.*, 1987; Forchhammer, 1995 ).

Symbiotic microbial fermentation accounts for 79 % of the dry matter digestion in muskoxen and is consequently the main source of energy for the animal (Barboza *et al.*, 2006). Only one study targeting polyadenylated eukaryotic mRNA in rumen samples from muskoxen fed a highly lignified diet has been performed thus far to characterize their microbiome (Qi *et al.*, 2011). Only the eukaryotic fraction was analysed in that study, describing a very high percentage of cellulolytic enzymes, but the presence of bacteria and archaea (3.4 and 0.1 % of total RNA reads, respectively) was also reported (Qi *et al.*, 2011). In the gut system, methanogenic archaea are the only micro-organisms responsible for methane production. Enteric methane emissions from muskoxen consuming brome hay comprised only 2.0–3.2 % of the gross energy intake (White & Lawler, 2002). Still, very little is known about the microbial ecosystem in the rumen and the hindgut of muskoxen. Considering the lack of knowledge regarding the microbial diversity in the digestive tract of this arctic ruminant, the present study aims to examine the diversity of archaea and bacteria in muskoxen faeces by applying a metagenomics approach. In addition, we include a comparison of the results obtained here with bacterial and archaeal datasets from other ruminant and non-ruminant herbivores to assess potential similarities/dissimilarities at a microbiota level.

## Methods

### Sampling.

**Fig. 1.**
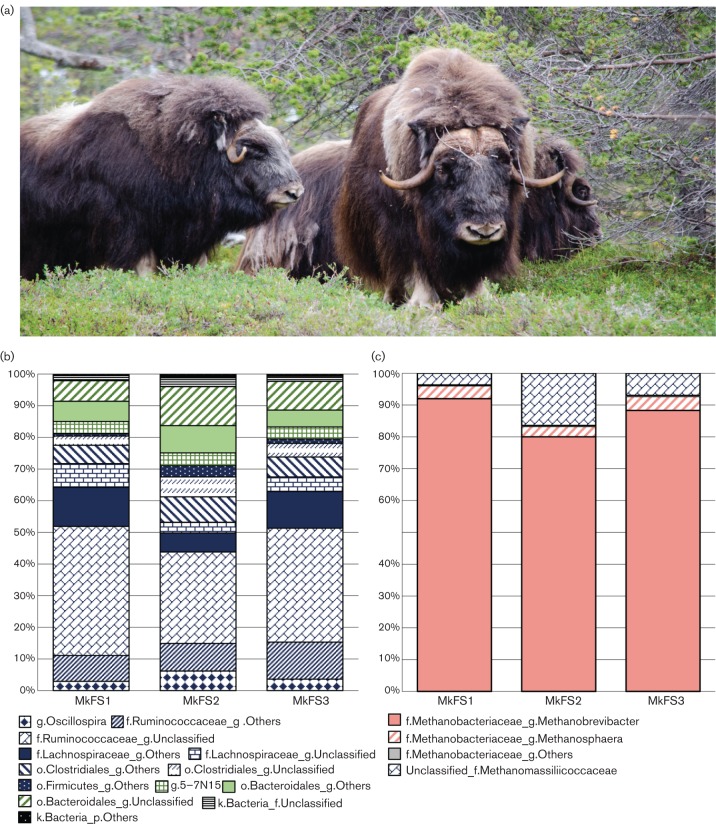
The faecal microbiota of muskoxen. Taxonomic classification of 16S rRNA sequences generated in this study was made using the RDP Classifier tool with a chimera-free curated 16S rRNA genomic databank. (a) Semi-domesticated muskoxen from the island of Ryøya whose faecal samples were used in this study. (b) Bar chart displaying bacterial taxonomy classification up to genus level. Broader colours refer to: *Firmicutes* (blue) and *Bacteroidetes* (green). (c) Proportion of sequences among the different archaeal genera. Red colours: class *Methanobacteria*; blue colours: *Methanoplasmatales*-related clones.

Previous studies have proven the reliability of using faeces as a proxy for describing the hindgut microbiome (Steelman *et al.*, 2012; de Oliveira *et al.*, 2013), at least for bacteria. Faecal samples [Muskoxen Faecal Sample (MkFS)1, MkFS2 and MkFS3] were collected immediately after dropping from three adult muskoxen grazing on patches of cultivated grassland and heather (September pasture) in open birch and pine forest on the island of Ryøya (69° 33′ N 18° 43′ E) near Tromsø, Norway (Fig. 1a). The animals belong to a herd of muskoxen, kept at Ryøya since the arrival of the first animals in 1979/1980, after originally being imported from East Greenland to an inland location in northern Norway (Blix *et al.*, 2011). Due to the territorial nature of this ruminant, samples were collected from a distance and therefore no gender or health status could be determined for each sample. The muskoxen faecal samples were immediately stored on ice and after no more than 3 h frozen at −80 °C prior to DNA extraction and molecular analysis.

### DNA extraction and PCR amplification.

DNA extraction was based on the protocol of the Repeated Bead Beating plus Column (RBB+C) method developed by Yu & Morrison (2004)), with minor modifications. DNA quantification was done using a NanoDrop 2000c spectrophotometer and solutions were stored at −20 °C until amplification by PCR.

PCR amplifications for *Bacteria* and *Archaea* were performed in an Eppendorf Mastercycler Gradient in 25 µl reaction volumes, with 12.5 µl of iProof High-Fidelity Master Mix (BioRad), 1 µl of each primer (400 nM), 1 µl of the corresponding DNA template and 1.25 µl DMSO to increase PCR efficiency.

Bacterial and archaeal 16S rRNA amplification was carried out with the bacterial primer set 27F and 515R (Pope *et al.*, 2012), giving an around 500 bp amplicon product; and the archaeal primer set 340F and 1000R (Gantner *et al.*, 2011), yielding an around 650 bp amplicon product. The reverse primer included an 8 nt Multiplex Identifier (MID) (Hamady *et al.*, 2008) for sample identification in downstream analysis. Both primer sets contained the Life Sciences primer A and B sequences necessary for pyrosequencing. PCRs were run with an initial denaturation step at 98 °C for 30 s; followed by 25/35 cycles (*Bacteria*/*Archaea*) consisting of 98 °C for 10 s, 60 °C/58 °C (*Bacteria*/*Archaea*) for 30 s and 72 °C for 45 s, completed with a final extension step at 72 °C for 7 min. Amplicon size was assessed by 1.5 % agarose gel and DNA concentration was quantified using a Qubit fluorimeter (Invitrogen). Sample products were then pooled in equimolar amounts, run in a 1 % agarose gel electrophoresis, excised and purified from the gel using NucleoSpin Gel and PCR Clean-up kit (Macherey-Nagel). The resulting DNA was stored at −20 °C until sequencing. PCR amplicons were sequenced with a 454/Roche GS FLX device using LIB-L titanium chemistry, at the Norwegian Sequencing Centre (NSC) in Oslo.

### Sequence processing and quality check.

Sequences for 16S rRNA genes from both microbial groups were analysed using the Quantitative Insights Into Microbial Ecology (QIIME) pipeline (Caporaso *et al.*, 2010a). Firstly, barcode and primer sequences were removed and sequences were discarded when: length was < 350 or > 650 nt; the number of homopolymer runs exceeded 6 nt; average quality score was below 25; and mismatches in primers occurred, thus ensuring high-quality sequences. The operational taxonomic unit (OTU)-clustering criterion was set on a 3 % genetic distance cutoff using the QIIME-incorporated version of usearch (Edgar, 2010), with a word length of 64 and discarding those OTUs below four reads. To avoid any potential bias at the OTU-clustering step, all sequences were trimmed to a similar 500 nt length. Chimeric sequences were identified using the uchime (Edgar *et al.*, 2011) tool in QIIME and discarding any sequence flagged as a putative chimera.

For interspecies comparisons between muskoxen faecal libraries and other datasets, the sequences were previously edited to obtain similar length and orientation. Muskoxen and Norwegian reindeer (Table S1, available with the online Supplementary Material) bacterial and archaeal libraries were reversed in orientation and we took the complementary sequence as sequencing was applied only on amplicons obtained from the reverse primer. In some instances, samples from the rumen were included for comparison among the bacterial libraries due to the scarcity of publicly available datasets from faeces or the lower intestine (Table S1). Samples with substantial differences in their total counts were finally included in the comparisons for the archaeal libraries. Accordingly, OTU clustering for these archaeal libraries included singletons (i.e. OTUs containing a single sequence) in the analyses to include as much microbial information as possible.

**Table 1.** Taxonomic classification of total shared OTUs by three muskoxen faecal samples Taxonomic identification was obtained using the RDP classifier tool incorporated in QIIME software with the RDP-II database. Numbers are displayed as a percentage of the total shared sequences per individual and the average of the three merged samples: MkFS1 (4982 sequences); MkFS2 (3398); MkFS3 (3651).

Phylum*	**Consensus lineage**	**MkFS1**	**MkFS2**	**MkFS3**	**Average**
F	k.Bacteria_p.Firmicutes_c.Clostridia_o.Clostridiales_f.Ruminococcaceae_g.Oscillospira	3.5	4.5	8.7	5.6
F	k.Bacteria_p.Firmicutes_c.Clostridia_o.Clostridiales_f.Ruminococcaceae_g.Ruminococcus	0.7	1.1	0.7	0.8
F	k.Bacteria_p.Firmicutes_c.Clostridia_o.Clostridiales_f.Ruminococcaceae_g.Others	8.6	10.6	6.6	8.6
F	k.Bacteria_p.Firmicutes_c.Clostridia_o.Clostridiales_f.Ruminococcaceae_g.Unclassified	39.3	32.1	24.9	32.1
F	k.Bacteria_p.Firmicutes_c.Clostridia_o.Clostridiales_f.Lachnospiraceae_g.Roseburia	1.7	2.2	0.6	1.5
F	k.Bacteria_p.Firmicutes_c.Clostridia_o.Clostridiales_f.Lachnospiraceae_g.Others	8.7	9.6	7.1	8.5
F	k.Bacteria_p.Firmicutes_c.Clostridia_o.Clostridiales_f.Lachnospiraceae_g.Unclassified	9.8	5.7	3.9	6.5
F	k.Bacteria_p.Firmicutes_c.Clostridia_o.Clostridiales_f.Others_g.Others	4.9	6.3	9.3	6.8
F	k.Bacteria_p.Firmicutes_c.Clostridia_o.Clostridiales_f.Unclassified	1.7	2.4	2.9	2.3
F	k.Bacteria_p.Firmicutes_c.Erysipelotrichi_o.Erysipelotrichales_f.Erysipelotrichaceae_g.Others	0.7	0.6	1.9	1.1
B	k.Bacteria_p.Bacteroidetes_c.Bacteroidia_o.Bacteroidales_f.Bacteroidaceae_g.5-7N15	4.5	4.8	5.2	4.8
B	k.Bacteria_p.Bacteroidetes_c.Bacteroidia_o.Bacteroidales_f.[Paraprevotellaceae]_g.CF231	0.9	2.5	2	1.8
B	k.Bacteria_p.Bacteroidetes_c.Bacteroidia_o.Bacteroidales_f.Prevotellaceae_g.Prevotella	1.1	0.6	1.5	1.1
B	k.Bacteria_p.Bacteroidetes_c.Bacteroidia_o.Bacteroidales_f.Rikenellaceae_g.Unclassified	2.6	2.2	2.1	2.3
B	k.Bacteria_p.Bacteroidetes_c.Bacteroidia_o.Bacteroidales_f.RF16_g.Unclassified	0.7	1.1	4	1.9
B	k.Bacteria_p.Bacteroidetes_c.Bacteroidia_o.Bacteroidales_f.Others_g.Others	5	2.6	8.3	5.3
B	k.Bacteria_p.Bacteroidetes_c.Bacteroidia_o.Bacteroidales_f.Unclassified	3.3	8.2	7.1	6.2
L	k.Bacteria_p.Lentisphaera_c.[Lentisphaeria]_o.Victivallales_f.Victivallaceae_g.Unclassified	0.2	0.7	0.3	0.4
O	k.Bacteria_p.Others_c.Others_o.Others_f.Others	1.1	1.6	1.7	1.5
O	k.Bacteria_p.Others_c.Others_o.Others_f.Unclassified	1	0.6	1.2	0.9

F, *Firmicutes;* B, *Bacteroidetes*; L, *Lentisphaera*; O, Other.

### Sequence analysis of bacterial and archaeal 16S rRNA genes.

The most abundant sequence for each OTU was taken as representative and subsequently aligned against the Greengenes core-set reference database using the Python-based version of the Nearest Alignment Space Termination (NAST) algorithm (Caporaso *et al.*, 2010b), with a minimum length of 150 nt and 75 % similarity cutoff. Taxonomic assignment down to the genus level for all the aligned chimera-free OTUs was performed using the RDP classifier tool (Cole *et al.*, 2003) in QIIME, which applies a Naïve-Bayesian algorithm on 8 k-mers at a default 80 % identity cutoff using the RDP-II database as reference taxonomy. Rank abundance plots evaluating sample richness and evenness were generated with the plot_rank_abundance_graph.py script in QIIME. Alpha-diversity analyses assessing species richness (Chao1) (Chao, 1984), evenness (Shannon-Wiener) (Shannon, 1948), total observed species and sample coverage (Good’s coverage) (Good, 1953) were performed on randomly subsampled datasets from each sample, and resulting rarefaction curves were obtained with the make_rarefaction_plots.py script. Pairwise sample dissimilarity analyses (beta diversity) were performed using unweighted UniFrac distance matrices (Lozupone & Knight, 2005) calculated with subsampled datasets adjusted to the sample yielding the lowest sequence counts. For interspecies comparisons between archaeal datasets from different animals, no rarefaction was performed due to the considerable differences in dataset size mentioned above. Principal coordinates for each dataset were calculated based on UniFrac distance matrices and principal coordinates analysis (PCoA) plots were generated. OTU network maps were created using the make_otu_network.py script in QIIME, and visualized with the Cytoscape (v3.1.1) platform (Shannon *et al.*, 2003). Statistical differences between pairs of sample datasets were assessed with unweighted UniFrac phylogenetic tree distances calculated based on iteration (Monte Carlo randomizations, 100 times) using the beta_significance.py script in QIIME. Heatmap analysis for comparisons with the different archaeal and bacterial datasets was done calculating the standard score (*z*-score) of the different phylotypes. Plots were generated with a customized version of the heatmap.2 script within the ‘gplots’ package in the ‘R’ software (R Development Core Team, 2008).

## Results and Discussion

### Bacterial diversity

**Fig. 2.**
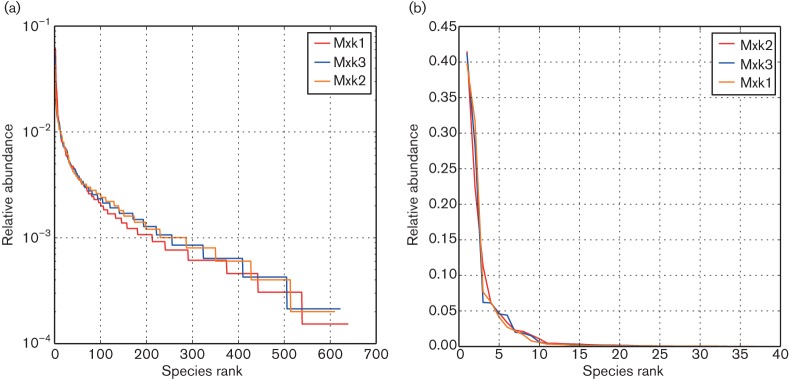
Rank abundance curves assessing sample richness and evenness in faeces of muskoxen. Curves show total species within a sample with species ranked from most (left) to least (right) abundant. (a) *Bacteria*; (b) *Archaea*.

A total of 16 209 500 bp-trimmed high-quality 16S rRNA gene sequences were obtained from the faeces of three adult muskoxen (MkFS1: 6 527; MkFS2: 4 987; MkFS3: 4 695). OTU clustering based on a 97 % similarity criterion resulted in 806 chimera-free OTUs, with 393 OTUs [74.2 % of the total sequences (12 031 seqs)] shared by the three animals (Fig. S1). These shared OTUs comprised 76.3 % (4 982 seqs), 68.1 % (3 398) and 77.7 % (3 651) of the total sequences in the MkFS1, MkFS2 and MkFS3 libraries, respectively (Table 1). Rank abundance plots showed steep curves for the three samples, thus indicating low sample evenness (Fig. 2a). Similar trends were also seen with rarefaction curves based on alpha diversity parameters (Fig. S2). Pairwise comparisons with unweighted UniFrac indicated statistical differences between the three samples (*P*<0.05) although not between MkFS1 and MkFS3 when corrected (*P*=0.078, Bonferroni corrected).

The bacterial communities were mostly dominated by *Firmicutes* (70.7–81.1 % of the total sequences) and *Bacteroidetes* (16.8–25.3 %), whereas *Tenericutes* (0.9–2.5 %) and* Cyanobacteria* (0.3–0.5 %) were also represented, albeit in minor proportions (Fig. 1b and Table S2). The *Firmicutes* and *Bacteroidetes* are also dominant in faeces from the cattle and horse hindgut (Dowd *et al.*, 2008; Steelman *et al.*, 2012). At family/genus level, *Ruminococcaceae* constituted the major *Firmicutes*-affiliated family, ranging from 43.8 to 51.9 % of the total sequences (Fig. 1b; Table S2). Around 28.9–40.9 % of the total sequences could not be designated to any characterized genus within this family. Of the shared sequences, uncharacterized genera belonging to *Ruminococcaceae* (24.9–39.3 %) and *Lachnospiraceae* (3.9–9.8 %) were dominant (Table 1). Several members related to *Ruminococcaceae* play a key role in the degradation of recalcitrant polysaccharides such as crystalline cellulose (Rincón *et al.*, 2001; Ben David *et al.*, 2015). Free-ranging muskoxen typically graze on highly lignified plants during winter, which may explain the high proportion of *Ruminococcaceae*-related constituents observed in this study (Fig. 1b and Table S2). Phylotypes affiliated to the family *Lachnospiraceae* constituted the second major group within *Firmicutes* (9.4–19.8 %), with *Roseburia* as the major genus (0.4–1.8 %). Uncharacterized genera within *Lachnospiraceae* constituted 5.2 % of total bacterial counts, on average (3.4–7.5 %). Several *Bacteroidetes*-related families such as *Bacteroidaceae* (3.8–4.2 %), *Rikenellaceae* (1.7–2.5 %) and *Prevotellaceae* (0.5–1.1 %) were also identified, with uncharacterized lineages affiliated to the order *Bacteroidales* accounting for 6.5–12.3 % of the total sequences (Fig. 1b and Table S2). Uncharacterized phylotypes within the order *Bacteroidales* accounted for 3.3–8.2 % of shared sequences (Table 1).

Overall, uncharacterized bacteria constituted 53.7–59.3 % of the total bacterial sequences. These relative proportions were higher than reported for steer fed diets with different fibre contents (Fernando *et al.*, 2010; De Oliveira *et al.*, 2013). A high proportion of novel bacterial phylotypes has also been described in the rumen of Norwegian and Svalbard reindeer (Pope *et al.*, 2012). Metatranscriptomics describing the eukaryotic fraction in the rumen of muskoxen reported a remarkable 17 % of carbohydrate active enzyme (CAZy) genes identified as ‘putative’ or ‘predictive proteins’, not found in any other rumen metagenome (Qi *et al.*, 2011). The high relative proportion of uncharacterized bacteria found in muskoxen faeces suggests the existence of novel bacterial phylotypes, which may be involved in the digestion of fibrous plants found at Arctic latitudes.

### Archaeal diversity

A total of 19 462 500 bp-trimmed high-quality 16S rRNA gene sequences were obtained from the three faecal samples (MkFS1: 6 320 sequences; MkFS2: 5 736; MkFS3: 7 406). OTU clustering gave 37 chimera-free OTUs, and 34 of these were shared by the three animals (99.1 % of the total sequences) (Fig. S1). Rank abundance plots showed low sample evenness and sequence abundance dominated by few archaeal phylotypes (Fig. 2b). This was corroborated by rarefaction curves based on several alpha diversity parameters (Fig. S2). *Euryarchaeota* was the only phylum detected in this study, with *Methanobacteriaceae* as the major family encompassing 83.6–96.3 % of the total sequences (Fig. 1c). Within this family, 80–92 % of the total sequences were designated to the genus *Methanobrevibacter* and 3.3–4.4 % as *Methanosphaera*-related phylotypes (Fig. 1c and Table S3). A dominance of *Methanobrevibacter* species is common in several other ruminant and non-ruminant herbivores in both rumen and faeces, without diet specificity (Sundset *et al.*, 2009a; Liu *et al.*, 2012; Turnbull *et al.*, 2012; Li *et al.*, 2014; Cersosimo *et al.*, 2015). High abundances of *Methanobrevibacter* species have been associated with high methane outputs (Wallace *et al.*, 2015). In contrast, muskoxen were reported to possess net methane emissions generally lower than for domesticated animals such as sheep and cattle (Blaxter & Clapperton, 1965; Johnson & Ward, 1996). The consumption of diets rich in plant secondary metabolites, such as the diet commonly eaten by muskoxen, may also negatively affect methanogenesis (Bodas *et al*., 2012). Considering that enteric methane is mostly produced in the rumen (Murray *et al.*, 1976), any potential estimation of the methanogenesis ratio for comparisons with other ruminants based solely upon archaeal community structure compositions from faeces should be attempted with caution.

Of the total sequences, 3.7–16.3 % were allocated to uncharacterized archaeal members of the family *Methanomassiliicoccaceae* (Fig. 1c and Table S3), representing the greatest source of variation between the samples; however, UniFrac-based beta diversity comparisons showed no statistical differences among the samples (*P*=1.0, Bonferroni corrected). This family belongs to group E2 within the class *Thermoplasmata* (Dridi *et al.*, 2012; Paul *et al.*, 2012). Several members of this class were also found at high proportion in other Arctic ruminants, such as Norwegian and Svalbard reindeer rumen (Sundset *et al.*, 2009a, b). Only one study has reported the presence of *Methanomassiliicoccaceae*-related phylotypes in the large intestine of herbivores (Luo *et al.*, 2013). In particular, group E2 methanogens produce methane mainly using methanol as carbon source (Gorlas *et al.*, 2012; Iino *et al.*, 2013). Methanol can be produced by the hydrolysis of methyl esters from pectin, and this can be degraded by genera associated with the *Bacteroides*,* Lachnospira* and *Ruminococcus* (Gradel & Dehority, 1972; Osborne & Dehority, 1989). The presence of *Methanomassiliicoccaceae*-related members in muskoxen faeces may partly be explained by the metabolism of methanol produced by fibre-degrading bacteria, mostly associated with the *Firmicutes*.

**Fig. 3.**
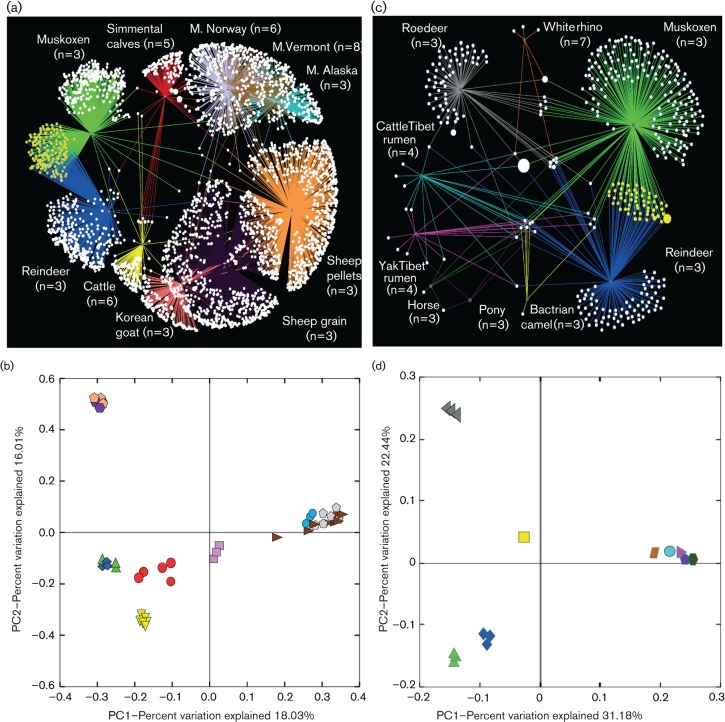
Molecular comparison between the faecal microbiota of muskoxen and datasets from other herbivores. (a, c) OTU network maps illustrating interactions between muskoxen bacterial (a) and archaeal (c) faecal microbiotas with other datasets. Radiating lines from each dot link OTUs to animal source. OTUs shared by muskoxen and reindeer are highlighted in yellow. (b, d) PCoA based on unweighted UniFrac for bacteria (b) and archaea (d). Sample colour and shape were allocated based on sample origin. (a, b) Muskoxen faeces (this study; triangle, green); Norwegian reindeer caecum (this study, only for comparisons; diamond, blue); Simmental calf faeces (circle, red); beef cattle faeces (triangle, yellow); Korean goat rumen (square, pink); Alaskan moose rumen (circle, turquoise), Norwegian moose rumen (pentagon, silver), Vermont moose rumen (triangle, brown); grain-diet sheep rumen (hexagon, purple); pellet-diet sheep rumen (pentagon, beige). (c, d) Muskoxen faeces (this study; triangle, green), Norwegian reindeer caecum (this study, only for comparisons; diamond, blue); Bactrian camel faeces (square, yellow); roe deer caecum (triangle, dark grey); white rhino hindgut (trapezoid, brown); pony faeces (pentagon, purple); horse faeces (hexagon, dark green); Tibetan cattle rumen (circle, light blue); Tibetan yak rumen (triangle, pink).

Comparative analyses were conducted between our muskoxen faecal bacterial and archaeal datasets with libraries from other ruminants to search for differences in their microbial traits (Fig. 3 and Table S1).

### Interspecies comparisons for bacterial libraries

**Fig. 4.**
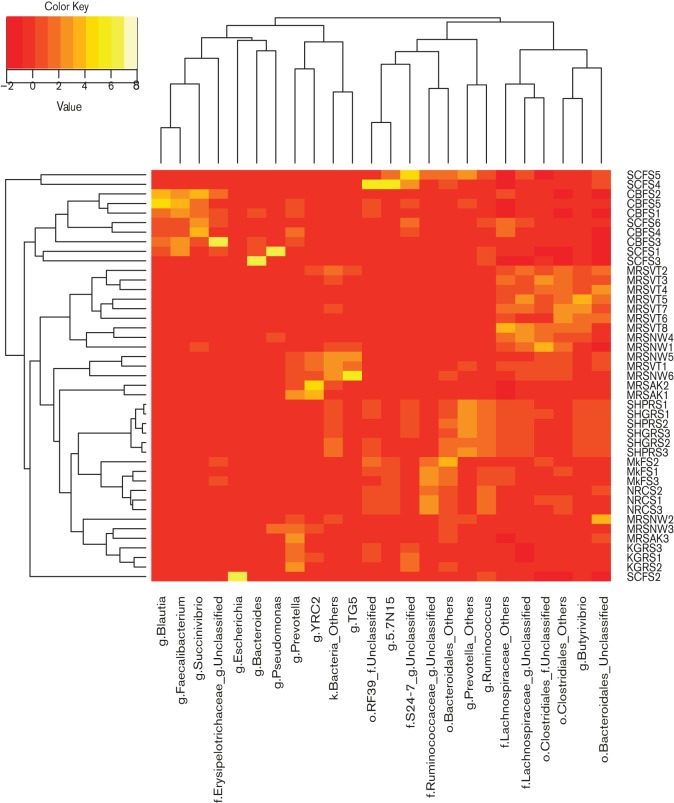
Heatmap analysis of the major bacterial phylotypes found in faecal or hindgut samples from different ruminants. Colour-coded profiles were created based on raw *z*-scores indicating the abundance of a particular phylotype in each sample. Hierarchical clustering was performed with calculated phylogenetic distances between samples based on their profiles. MkFS, muskoxen faeces ; NRCS, Norwegian reindeer caecum; CBFS, cattle beef faeces; SCFS, Simmental calf faeces; KGRS, Korean goat rumen; MRSAK, moose rumen Alaska; MRSNW, moose rumen Norway; MRSVT, moose rumen Vermont; SHGRS, sheep rumen grain diet; SHPRS, sheep rumen pellet diet.

OTU Network mapping and PCoA (Fig. 3a,b) showed a higher number of bacterial OTUs shared between the muskoxen and reindeer datasets compared with other ruminants included in the analysis. Shannon index values were higher in muskoxen and reindeer libraries than in the other datasets, except rumen samples from sheep (fed grains or pellets) (Kittelman *et al.*, 2013) (Fig. S3). Sampling site (faeces, caecum or rumen) also had a stronger influence on sample clustering with no significant differences observed between muskoxen faeces, reindeer caecum and cattle faeces (Durso *et al*., 2010;Klein-Jöbstl *et al*., 2014-)(Table S4). Instead, when considering the libraries together, only reindeer libraries did not show statistical differences from muskoxen (Table S4). OTU heatmap analysis with hierarchical clustering based on *z*-score profiles of the major bacterial phyla from each animal supported these findings, showing sub-clustering with samples from muskoxen and reindeer (Fig. 4). These results suggest that muskoxen and reindeer possessed comparable bacterial profiles. Despite the differences in diet (muskoxen: pasture; reindeer: grain-based diet), these animals shared a similar Arctic environment, which may account for their similar bacterial profiles; however, the dataset from moose from northern Norway was different from that from muskoxen. The moose samples were originally collected from the rumen instead of the caecum or faeces (Ishaq & Wright, 2014). The difference in sampling site would partly explain the dissimilarities of moose libraries with those from ruminants co-habiting similar environments (muskoxen and reindeer). The same was observed for other animals phylogenetically related to muskoxen, such as sheep or goats (Lee *et al*., 2012; Kittelmann *et al*., 2013)(Table S4). Sampling site has been shown to greatly influence microbial profiles (de Oliveira *et al.*, 2013). Future comparative analysis should consider the effects produced by this parameter on interpretation of the results.

### Interspecies comparisons for archaeal libraries

**Fig. 5.**
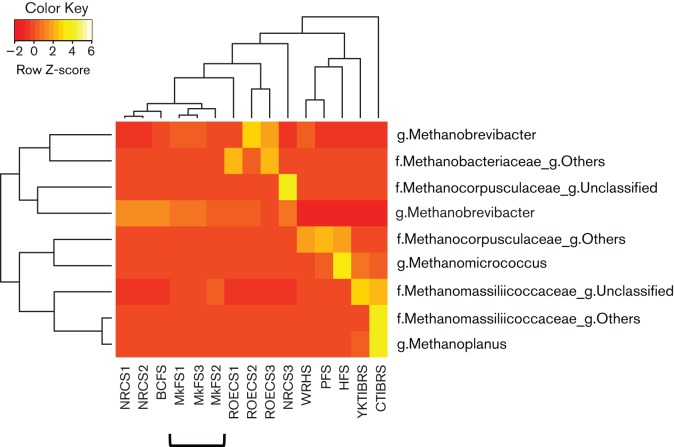
Heatmap analysis showing the distribution of the major archaeal phylotypes in faecal or hindgut samples from different herbivores. Colour-coded profiles were created based on raw *z*-scores indicating the abundance of a particular phylotype in each sample. Hierarchical clustering was performed with calculated distances between samples based on their profiles. MkFS, muskoxen faeces; NRCS, Norwegian reindeer caecum; ROECS, roe deer caecum; BCFS, Bactrian camel faeces; HFS, horse faeces; PFS, pony faeces; WRHS, white rhino hindgut; CTIBRS, cattle Tibet rumen; YKTIBRS, yak Tibet rumen.

OTU Network mapping revealed a higher degree of OTUs shared by muskoxen and Norwegian reindeer fed with pellets concentrate (Fig. 3c). Unweighted UniFrac-based PCoA plots also displayed closer clustering for samples from these two Arctic ruminants (Fig. 3d), with overall diversity being higher in both datasets compared with other libraries (Fig. S4). In contrast, OTU-Heatmap analysis resulted in muskoxen faecal samples branching separately (Fig. 5). UniFrac-based statistical tests with libraries treated separately or together corroborated the differences observed between the two Arctic ruminants (*P*=1.0e-02, Bonferroni corrected) (Table S4). Unexpectedly, no statistical differences were found between muskoxen faeces and samples from hindgut fermenters such as horse, pony and white rhinoceros (*P*>0.05). These libraries were obtained with samples collected from animals fed diets constituted by a high proportion of fibre supplemented with concentrated feed similar to that for muskoxen, and different from that for reindeer (grains). Nonetheless, *Methanocorpusculum labreanum*-related phylotypes were dominant in these hindgut fermenters (Luo *et al.*, 2013; Lwin & Matsui, 2014), whereas no representatives of *Methanomicrobia* were found in muskoxen. The libraries from hindgut fermenters possessed substantially lower sequence counts compared with muskoxen (Table S1), with lower sequencing depths; however, a small sample size was also observed in rumen dataset from Tibetan yak and cattle (Huang *et al*., 2012) (Table S1), which yielded statistical differences with muskoxen libraries (Table S6). Whether sequencing depth may have driven the lack of statistical differences observed among these datasets (muskoxen and hindgut fermenters) remains to be clarified.

This study is the first investigation into the faecal microbiome structure in the muskoxen. The large number of uncharacterized bacteria described here along with previous reports of novel features described for the eukaryotic fraction in their rumen microbiome (Qi *et al.*, 2011) emphasizes the potential for the bacterial microbiome housed by this Arctic ruminant. Interest in the mining of these novel enzymes involved in polysaccharide degradation is also conceivable, given the high efficiency of these microbiomes to deconstruct low-quality forages and enabling the host to survive under austere nutritional conditions.

## Supplementary Data

3117 Supplementary File 1
