# Supplementary material for: First insight into the faecal microbiota of the high Arctic muskoxen (Ovibos moschatus)
Source: Microb Genom. 2016 Jul 29;2(7):e000066. doi: 10.1099/mgen.0.000066 (PMC5343138; doi:10.1099/mgen.0.000066)
Supplement: Supplementary file 1 [file mgen-02-66-s001.pdf]

Table S1.

Sample ID	Animal	Sampling site	No. sequences	Microbe targeted	Reference	Accession number
MkFS	Muskoxen	Feces	18020/20845	Bacteria/Archaea	This study	SRA: SRP049372
NRCS	Norwegian reindeer	Cecum	12235 /14126	Bacteria/Archaea	This study	*SRA: SRP063699
CBFS	Cattle beef	Feces	7974	Bacteria	Durso et al. 2010	FJ672948- FJ674268/ FJ675665- FJ685516
SCFS	Simmental cattle calves	Feces	45075	Bacteria	Klein-Jöbstl et al. 2014	SRA: PRJEB4554
KGRS	Korean goat	Rumen	5019	Bacteria	Lee et al. 2012	SRA: SRR497631
SHGRS	Sheep rumen grain diet	Rumen	240109	Bacteria	Kittelman et al. 2013	EBI: ERP002014
SHPRS	Sheep rumen pellets diet	Rumen	224072	Bacteria	Kittelman et al. 2013	EBI: ERP002014
MRSAK	Moose rumen Alaska	Rumen	17245	Bacteria	Ishaq et al. 2014	SRA: SRP022590
MRSNW	Moose rumen Northern Norway	Rumen	22937	Bacteria	Ishaq et al. 2014	SRA: SRP022590
MRSVT	Moose rumen Vermont	Rumen	10703	Bacteria	Ishaq et al. 2014	SRA: SRP022590
ROECS	Roe deer	Cecum	191404	Archaea	Li et al. 2014	EU458114-EU475873/ EU771093-EU779492
BCFS	Bactrian camel	Feces	35	Archaea	Turnbull et al. 2012	HQ659060-HQ659099
HF	Horse	Feces	50	Archaea	Lwin and Matsui 2014	AB739303–AB739402
PF	Pony	Feces	49	Archaea	Lwin and Matsui 2014	AB739303–AB739402
WRHS	White rhino	Hindgut	47	Archaea	Luo et al. 2013	JX833566-JX833612
CTIBRS	Cattle Tibet	Rumen	113	Archaea	Huang et al. 2012	JF807063-JF807176
YKTIBRS	Yak Tibet	Rumen	128	Archaea	Huang et al. 2012	JF807177-JF807311

Durso et al., 2010. Appl Environ Microbiol 76:4858-4862. doi: 10.1128/AEM.00207-10
Klein-Jöbstl et al., 2014. Front Microbiol 5:622. doi: 10.3389/fmicb.2014.00622
Lee et al., 2012. Appl Environ Microbiol. 5983-5993. doi: 10.1128/AEM.00104-12
Kittelman et al., 2013. PLoS One. 8:e47879. doi: 10.1371/journal.pone.0047879

Ishaq et al., 2014. Microb Ecol. 68:185-195. doi: 10.1007/s00248-014-0399-0
Li et al., 2014. PLoS One 9:e114513. doi: 10.1371/journal.pone.0114513
Lwin & Matsui 2014. Archaea doi: 10.1155/2014/483574
Luo et al., 2013. BMC Microbiol 13:207. doi: 10.1186/1471-2180-13-207
Turnbull et al., 2012. Res Vet Sci 93:246-249. doi: 10.1016/j.rvsc.2011.08.013
*Manuscript under revision for publication elsewhere

Figure S1.

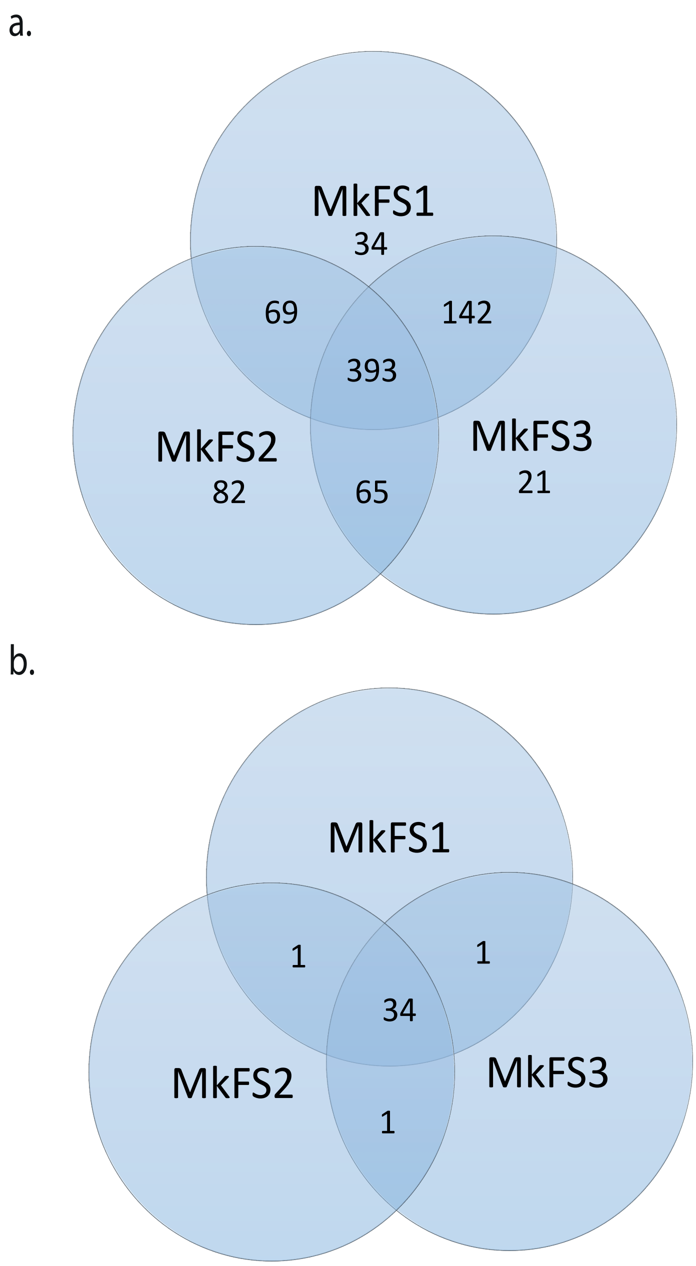
a.
MkFS1
34
69
142
393
MkFS2
82
65
MkFS3
21
b.
MkFS1
1
1
34
MkFS2
1
MkFS3

Figure S2.

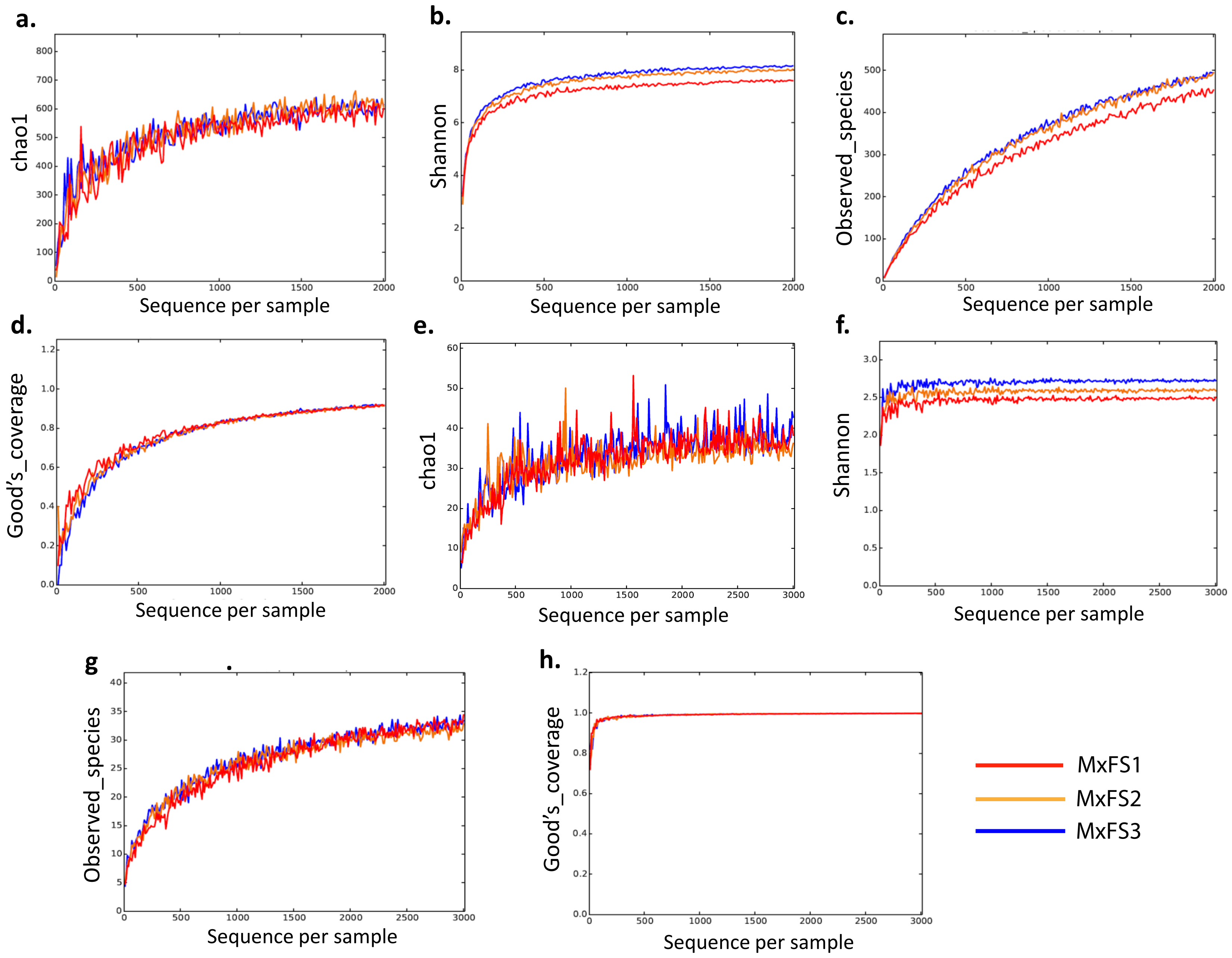
a.
chao1
Sequence per sample
b.
Shannon
Sequence per sample
c.
Observed_species
Sequence per sample
d.
Good's_coverage
Sequence per sample
e.
chao1
Sequence per sample
f.
Shannon
Sequence per sample
g.
Observed_species
Sequence per sample
h.
Good's_coverage
Sequence per sample
MxFS1
MxFS2
MxFS3

Table S2.

PHYLUM	MkFS1	MkFS2	MkFS3	Average
p.Firmicutes	81.1	70.7	78.7	76.8
p.Bacteroidetes	16.8	25.3	18.2	20.1
p.Tenericutes	0.9	2.5	0.9	1.4
p.Cyanobacteria	0.5	0.3	0.6	0.5
p.Lentisphaerae	0.2	0.3	1.1	0.5
k.Bacteria_Others	0.5	0.9	0.5	0.6
**TOTAL**	100	100	100	100

CLASS	MkFS1	MkFS2	MkFS3	Average
p.Firmicutes_c.Clostridia	80.4	68.3	78	75.6
p.Firmicutes_c.Erysipelotrichi	0.7	1.7	0.6	1
p.Firmicutes_c.Bacilli	0	0.4	0	0.1
p.Bacteroidetes_c.Bacteroidia	16.5	25	18.1	19.9
p.Bacteroidetes_Others	0.3	0.3	0.1	0.2
p.Tenericutes_c.Mollicutes	0.9	2.3	0.5	1.2
p.Tenericutes_c.RF3	0	0.2	0.3	0.2
p.Cyanobacteria_c.4C0d-2	0.5	0.3	0.6	0.5
p.Lentisphaerae_c.Lentishphaeria	0.2	0.3	1.1	0.5
k.Bacteria_Others	0.5	1.2	0.7	0.8
**TOTAL**	**100**	**100**	**100**	**100**

ORDER	MkFS1	MkFS2	MkFS3	Average
c.Clostridia_o.Clostridiales	80.4	68.3	78	75.6
c.Erysipelotrichi_o.Erysipelotrichales	0.7	1.7	0.6	1
c.Bacilli_o.Bacillales	0	0.4	0	0.1
c.Bacteroidia_o.Bacteroidales	16.5	25	18.1	19.9
p.Bacteroidetes_Others	0.3	0.3	0.1	0.2
c.Mollicutes_o.RF39	0.9	2.3	0.5	1.2
p.Tenericutes_Others	0	0.2	0.3	0.2
p.Cyanobacteria_c.4C0d-2_o.YS2	0.5	0.3	0.6	0.5
c.Lentispaheria_o.Victivallales	0.2	0.3	1.1	0.5
k.Bacteria_Others	0.5	1.2	0.7	0.8
**TOTAL**	**100**	**100**	**100**	**100**

FAMILY	MkFS1	MkFS2	MkFS3	Average
o.Clostridiales_f.Ruminococcaceae	51.9	44	51.3	49.1
o.Clostridiales_f.Lachnospiraceae	20.1	10.3	16.6	15.7
o.Clostridiales_f.Veillonellaceae	0.3	0.7	0.4	0.5
o.Clostridiales_f.Others	5.5	7.9	5.8	6.4
o.Clostridiales_g.Unclassified	2.4	5.1	3.5	3.7
c.Erysipelotrichales_f.Erysipelotrichaceae	0.7	1.7	0.6	0.2
p.Firmicutes_f.Others	0	0.3	0.3	0.2
o.Bacteroidales_f.Bacteroidaceae	4.2	5.1	3.8	4.4
o.Bacteroidales_f.Rikenellaceae	2	2.5	1.7	2.1
o.Bacteroidales_f.Paraprevotellaceae	1	1.8	2.3	1.7
o.Bacteroidales_f.RF16	0.6	2.8	0.8	1.4
o.Bacteroidales_f.Prevotellaceae	0.9	1.1	0.5	0.8
o.Bacteroidales_f.Others	5	6.4	2.5	4.6
Unclassified_o.Bacteroidales	2.8	5.5	6.6	5
p.Bacteroidetes_f.Others	0.3	0.3	0.1	0.2
c.Mollicutes_Unclassified_o.RF39	0.9	2.3	0.2	1.1
c.Mollicutes_f.Others	0.2	0.4	0.6	0.4
p.Cyanobacteria_o.YS2_f.Unclassified	0.5	0.3	0.6	0.5
c.Lentispaheria_o.Victivallales_f.Victivallaceae0.2	0.2	0.3	1.1	0.5
k.Bacteria_Others	0.5	1.2	0.7	0.8
**TOTAL**	**100**	**100**	**100**	**100**

GENUS	MkFS1	MkFS2	MkFS3	Average
**f.Ruminococcaceae_g.Oscillospira**	2.9	6.2	3.6	4.2
**f.Ruminococcaceae_g.Ruminococcus**	0.8	0.6	1.9	1.1
**f.Ruminococcaceae_g.Others**	7.4	8.1	9.8	8.4
**f.Ruminococcaceae_g.Unclassified**	40.8	28.9	36	35.2
**f.Lachnospiraceae_g.Roseburia**	1.4	0.4	1.8	1.2
**f.Lachnospiraceae_g.Anaerostipes**	1.5	0.5	0.4	0.8
**f.Lachnospiraceae_g.Others**	9.4	5.1	9.4	8
**f.Lachnospiraceae_g.Unclassified**	7.5	3.4	4.6	5.2
**o.Clostridiales_g.Others**	5.8	8	6.3	6.7
**o.Clostridiales_g.Unclassified**	2.9	6.3	4.3	4.5
**f.Erysipelotrichaceae_g.Others**	0.6	1.2	0.5	0.8
**p.Firmicutes_g.Others**	0.2	2.3	0.9	1.1
**f.Bacteroidaceae_g.5-7N15**	3.8	4.1	3.8	3.9
**f.Prevotellaceae_g.Prevotella**	0.9	1.1	0.5	0.8
**f.Paraprevotellaceae_g.CF231**	0.7	1.4	1.9	1.3
**o.Bacteroidales_Others**	4.8	6.1	2.9	4.6
**o.Bacteroidales_g.Unclassified**	6.5	12.3	9.1	9.3
**c.Mollicutes_o.RF39_f.Unclassified**	0.9	2.3	0.2	1.1
**p.Cyanobacteria_o.YS2_f.Unclassified**	0.5	0.3	0.6	0.5
**f.Victivallaceae_g.Unclassified**	0.2	0.2	0.8	0.4
**k.Bacteria_Others**	0.5	1.2	0.7	0.8
**TOTAL**	**100**	**100**	**100**	**100**

	MkFS1	MkFS2	MkFS3	Average
**Uncharacterized**	59.33	53.7	55.6	56.2

TOTAL sequences: MkFS1 (6.527); MkFS2 (4.987); MkFS3 (4.695)

Table S3.

Nearest valid representative (RDP-II)	MxFS1 (% total sequences: 6,320)	MxFS2 (% total sequences: 5,736)	MxFS3 (% total sequences: 7,406)
f.*Methanobacteriaceae*_g.*Methanobrevibacter*	92.0	80	88.3
f.*Methanobacteriaceae*_g.*Methanosphaera*	4.1	3.3	4.4
f.*Methanobacteriaceae*_Others	0.2	0.3	0.3
f.*Methanomassilicoccaceae*_g.Unclassified	3.7	16.4	7.0

Figure S3.

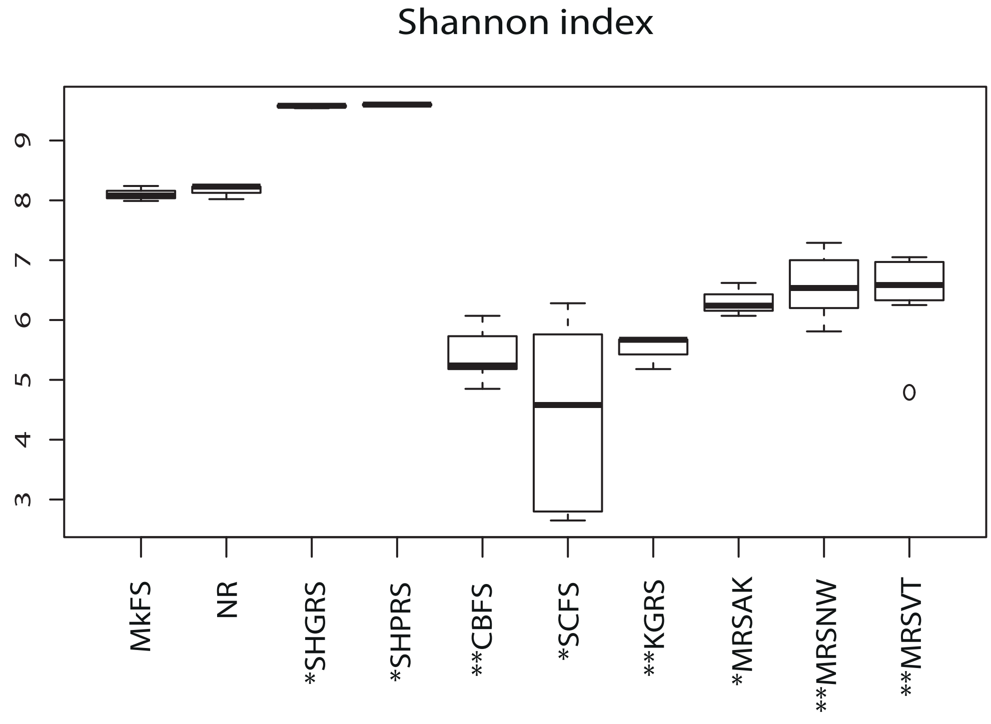
Shannon index
3
4
5
6
7
8
9
MkFS
NR
*SHGRS
*SHPRS
**CBFS
*SCFS
**KGRS
*MRSAK
**MRSNW
**MRSVT

Table S4.

Bacteria (Separated by sample):

Sample 1	Sample 2	*p*-value	*p*-value(Bonferroni corrected)
MkFS1	CBFS1	0.00	0.01
MkFS2	CBFS1	0.04	1.00
MkFS3	CBFS1	0.11	1.00
MkFS1	CBFS2	0.09	1.00
MkFS2	CBFS2	0.02	1.00
MkFS3	CBFS2	0.23	1.00
MkFS1	CBFS3	0.00	0.01
MkFS2	CBFS3	0.00	0.01
MkFS3	CBFS3	0.00	0.01
MkFS1	CBFS4	0.01	1.00
MkFS2	CBFS4	0.00	0.01
MkFS3	CBFS4	0.03	1.00
MkFS1	CBFS5	0.03	1.00
MkFS2	CBFS5	0.03	1.00
MkFS3	CBFS5	0.16	1.00
MkFS1	CBFS6	0.21	1.00
MkFS2	CBFS6	0.36	1.00
MkFS3	CBFS6	0.70	1.00
MkFS1	KGRS1	0.00	0.01
MkFS2	KGRS1	0.00	0.01
MkFS3	KGRS1	0.00	0.01
MkFS1	KGRS2	0.00	0.01
MkFS2	KGRS2	0.00	0.01
MkFS3	KGRS2	0.00	0.01
MkFS1	KGRS3	0.00	0.01
MkFS2	KGRS3	0.01	1.00
MkFS3	KGRS3	0.00	0.01
MkFS1	MRSAK1	0.00	0.01
MkFS2	MRSAK1	0.00	0.01
MkFS3	MRSAK1	0.00	0.01
MkFS1	MRSAK2	0.00	0.01
MkFS2	MRSAK2	0.00	0.01
MkFS3	MRSAK2	0.00	0.01
MkFS1	MRSAK3	0.00	0.01
MkFS2	MRSAK3	0.00	0.01
MkFS3	MRSAK3	0.00	0.01
MkFS1	MRSNW1	0.00	0.01
MkFS2	MRSNW1	0.00	0.01
MkFS3	MRSNW1	0.00	0.01
MkFS1	MRSNW2	0.00	0.01
MkFS2	MRSNW2	0.00	0.01

MkFS3	MRSNW2	0.00	0.01
MkFS1	MRSNW3	0.00	0.01
MkFS2	MRSNW3	0.00	0.01
MkFS3	MRSNW3	0.00	0.01
MkFS1	MRSNW4	0.00	0.01
MkFS2	MRSNW4	0.00	0.01
MkFS3	MRSNW4	0.00	0.01
MkFS1	MRSNW5	0.00	0.01
MkFS2	MRSNW5	0.00	0.01
MkFS3	MRSNW5	0.00	0.01
MkFS1	MRSNW6	0.00	0.01
MkFS2	MRSNW6	0.00	0.01
MkFS3	MRSNW6	0.00	0.01
MkFS1	MRSVT1	0.00	0.01
MkFS2	MRSVT1	0.00	0.01
MkFS3	MRSVT1	0.00	0.01
MkFS1	MRSVT2	0.00	0.01
MkFS2	MRSVT2	0.00	0.01
MkFS3	MRSVT2	0.00	0.01
MkFS1	MRSVT3	0.00	0.01
MkFS2	MRSVT3	0.00	0.01
MkFS3	MRSVT3	0.00	0.01
MkFS1	MRSVT4	0.00	0.01
MkFS2	MRSVT4	0.00	0.01
MkFS3	MRSVT4	0.00	0.01
MkFS1	MRSVT5	0.00	0.01
MkFS2	MRSVT5	0.00	0.01
MkFS3	MRSVT5	0.00	0.01
MkFS1	MRSVT6	0.55	1.00
MkFS2	MRSVT6	0.87	1.00
MkFS3	MRSVT6	0.71	1.00
MkFS1	MRSVT7	0.00	0.01
MkFS2	MRSVT7	0.00	0.01
MkFS3	MRSVT7	0.00	0.01
MkFS1	MRSVT8	0.00	0.01
MkFS2	MRSVT8	0.00	0.01
MkFS3	MRSVT8	0.00	0.01
MkFS1	NRCS1	0.37	1.00
MkFS2	NRCS1	0.00	0.01
MkFS3	NRCS1	0.09	1.00
MkFS1	NRCS2	0.96	1.00
MkFS2	NRCS2	0.87	1.00
MkFS3	NRCS2	0.74	1.00
MkFS1	NRCS3	1.00	1.00
MkFS2	NRCS3	0.84	1.00

MkFS3	NRCS3	0.85	1.00
MkFS1	SCFS1	0.15	1.00
MkFS2	SCFS1	0.11	1.00
MkFS3	SCFS1	0.55	1.00
MkFS1	SCFS2	0.00	0.01
MkFS2	SCFS2	0.00	0.01
MkFS3	SCFS2	0.00	0.01
MkFS1	SCFS3	0.00	0.01
MkFS2	SCFS3	0.00	0.01
MkFS3	SCFS3	0.00	0.01
MkFS1	SCFS4	0.10	1.00
MkFS2	SCFS4	0.05	1.00
MkFS3	SCFS4	0.01	1.00
MkFS1	SCFS5	0.03	1.00
MkFS2	SCFS5	0.00	1.00
MkFS3	SCFS5	0.00	0.01
MkFS1	SHGRS1	0.00	0.01
MkFS2	SHGRS1	0.00	0.01
MkFS3	SHGRS1	0.00	0.01
MkFS1	SHGRS2	0.00	0.01
MkFS2	SHGRS2	0.00	0.01
MkFS3	SHGRS2	0.00	0.01
MkFS1	SHGRS3	0.00	0.01
MkFS2	SHGRS3	0.00	0.01
MkFS3	SHGRS3	0.00	0.01
MkFS1	SHPRS1	0.00	0.01
MkFS2	SHPRS1	0.00	0.01
MkFS3	SHPRS1	0.00	0.01
MkFS1	SHPRS2	0.00	0.01
MkFS2	SHPRS2	0.00	0.01
MkFS3	SHPRS2	0.00	0.01
MkFS1	SHPRS3	0.00	0.01
MkFS2	SHPRS3	0.00	0.01
MkFS3	SHPRS3	0.00	0.01

Bacteria (Samples altogether):

**Sample 1**	**Sample 2**	***p*-value**	***p*-value(Bonferroni corrected)**
MkFS	NRCS	0.51	1.00
MkFS	SCFS	0.00	0.01
MkFS	CBFS	0.00	0.01
MkFS	KGRS	0.00	0.01
MkFS	MRSAK	0.00	0.01
MkFS	MRSNW	0.00	0.01
MkFS	MRSVT	0.00	0.01

MkFS	SHGRS	0.00	0.01
MkFS	SHRPS	0.00	0.01

Archaea (Separated by sample):

**Sample 1**	**Sample 2**	***p*-value**	***p*-value(Bonferroni corrected)**
MkFS1	NRCS1	0.00	0.01
MkFS2	NRCS1	0.00	0.01
MkFS3	NRCS1	0.00	0.01
MkFS1	NRCS2	0.00	0.01
MkFS2	NRCS2	0.00	0.01
MkFS3	NRCS2	0.00	0.01
MkFS1	NRCS3	0.00	0.01
MkFS2	NRCS3	0.00	0.01
MkFS3	NRCS3	0.00	0.01
MkFS1	ROECS1	0.00	0.01
MkFS2	ROECS1	0.00	0.01
MkFS3	ROECS1	0.00	0.01
MkFS1	ROECS2	0.00	0.01
MkFS2	ROECS2	0.00	0.01
MkFS3	ROECS2	0.00	0.01
MkFS1	ROECS3	0.00	0.01
MkFS2	ROECS3	0.00	0.01
MkFS3	ROECS3	0.00	0.01
MkFS1	BCFS	0.00	0.01
MkFS2	BCFS	0.00	0.01
MkFS3	BCFS	0.00	0.01
MkFS1	CTIBRS	0.00	0.01
MkFS2	CTIBRS	0.00	0.01
MkFS3	CTIBRS	0.00	0.01
MkFS1	WRHS	0.38	1.00
MkFS2	WRHS	0.38	1.00
MkFS3	WRHS	0.29	1.00
MkFS1	YKTIBRS	0.00	0.01
MkFS2	YKTIBRS	0.00	0.01
MkFS3	YKTIBRS	0.00	0.01
MkFS1	PFS	0.57	1.00
MkFS2	PFS	0.50	1.00
MkFS3	PFS	0.29	1.00
MkFS1	HFS	0.10	1.00
MkFS2	HFS	0.13	1.00
MkFS3	HFS	0.18	1.00
MkFS1	MkFS2	0.00	0.01
MkFS2	MkFS3	0.00	0.01
MkFS3	MkFS3	0.00	0.01

Archaea (Samples altogether):

**Sample 1**	**Sample 2**	***p*-value**	***p*-value(Bonferroni corrected)**
MkFS	NRCS	0.00	0.00
MkFS	BCFS	0.00	0.00
MkFS	HFS	0.01	0.32
MkFS	PFS	0.19	1.00
MkFS	ROECS	0.00	0.00
MkFS	WRHS	0.12	1.00
MkFS	YKTIBRS	0.00	0.00
MkFS	CTIBRS	0.00	0.00

Figure S4.

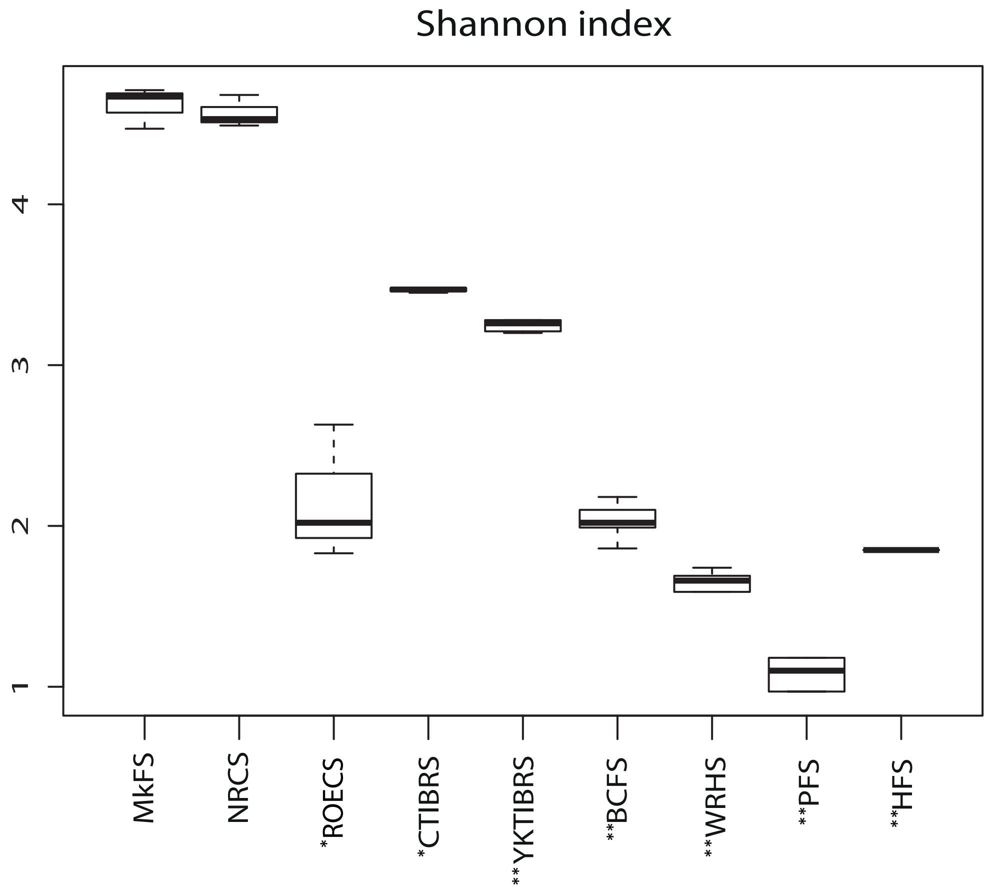
Shannon index
1
2
3
4
MkFS
NRCS
*ROECS
*CTIBRS
**YKTIBRS
**BCFS
**WRHS
**PFS
**HFS
